# Higher number of multidisciplinary tumor board meetings per case leads to improved clinical outcome

**DOI:** 10.1186/s12885-020-06809-1

**Published:** 2020-04-28

**Authors:** Marius Freytag, Ulrich Herrlinger, Stefan Hauser, Franz G. Bauernfeind, Maria A. Gonzalez-Carmona, Jennifer Landsberg, Jens Buermann, Hartmut Vatter, Tobias Holderried, Thorsten Send, Martin Schumacher, Arne Koscielny, Georg Feldmann, Mario Heine, Dirk Skowasch, Niklas Schäfer, Benjamin Funke, Michael Neumann, Ingo G. H. Schmidt-Wolf

**Affiliations:** 1 Department of Integrated Oncology, Center of Integrated Oncology ABCD, University Hospital Bonn, Venusberg-Campus 1, 53127 Bonn, Germany; 2 Department of Neurology, University Hospital Bonn, Bonn, Germany; 3 Department of Urology, University Hospital Bonn, Bonn, Germany; 4 Department of Internal Medicine III, University Hospital Bonn, Bonn, Germany; 5 Department of Internal Medicine I, University Hospital Bonn, Bonn, Germany; 6 Department of Dermatology, University Hospital Bonn, Bonn, Germany; 7 Department of General Surgery, University Hospital Bonn, Bonn, Germany; 8 Department of Neurosurgery, University Hospital Bonn, Bonn, Germany; 9 Department of Otorhinolaryngology, University Hospital Bonn, Bonn, Germany; 10 Department of Internal Medicine II, University Hospital Bonn, Bonn, Germany

**Keywords:** Multidisciplinary tumor board, Cancer, Overall survival, Relapse free survival, Time to progression, Matched pair analysis

## Abstract

**Background:**

This analysis aims at evaluating the impact of multidisciplinary tumor boards on clinical outcome of multiple tumor entities, the effect of the specific number of multidisciplinary tumor boards and potential differences between the tumor entities.

**Methods:**

By a matched-pair analysis we compared the response to treatment, overall survival, relapse or disease free survival and progression free survival of patients whose cases were discussed in a tumor board meeting with patients whose cases were not. It was performed with patients registered in the cancer registry of the University of Bonn and diagnosed between 2010 and 2016. After the matching process with a pool of 7262 patients a total of 454 patients with 66 different tumor types were included in this study.

**Results:**

First, patients with three or more multidisciplinary tumor board meetings in their history show a significantly better overall survival than patients with no tumor board meeting. Second, response to treatment, relapse free survival and time to progression were not found to be significantly different. Third, there was no significant difference for a specific tumor entity.

**Conclusion:**

This study revealed a positive impact of a higher number of multidisciplinary tumor boards on the clinical outcome. Also, our analysis hints towards a positive effect of multidisciplinary tumor boards on overall survival.

## Background

Despite the fast advancing diagnostic possibilities and treatment methods, cancer with being responsible for 25.3% of all deaths in 2016 is still the second largest cause of death in Germany ^1^. An effort to further improve the outcome was to introduce multidisciplinary tumor board meetings, where physicians specialized in medical oncology, surgery, radiology or various other fields related to the patient’s tumor come together to discuss and agree on the best individual diagnostic and treatment plan. Between 2010 and 2016 a total of 1512 in ODSeasy documented multidisciplinary tumor board (MTB) meetings were held at the University of Bonn. While the number of tumor boards continued to grow over this period, it is important to evaluate the benefit of this effort for the patient. Previous studies were able to show improvement of survival for some specific tumor entity groups like lung cancer [1–3], head and neck cancer [4, 5], hepatocellular cancer [6, 7] and breast cancer [8] while some other studies did not show an significant improvement of survival for entities like pancreatic cancer [9] colorectal cancer [10], multiple myelomas [11] or metastatic germ cell tumors [12]. Analyses which tried to draw the big picture by taking multiple entities into consideration via metanalysis [13] or general comparison of cancer centers with and without multidisciplinary tumor board meetings [14] showed little to no evidence of an improved clinical outcome. This analysis includes matched patients of 66 different tumor types to further evaluate the clinical outcome of multidisciplinary tumor board meetings and investigate the influence of the number of these case discussions per patient.

## Methods

### Patient data

All patient data was collected from ODS (Oncologic documentation system) easy net, the CIO (Center for Integrated Oncology) cancer registry of the University of Bonn which contains all tumor types except gynecological tumors like breast or ovarian carcinomas. The 7262 patients that were diagnosed between the 1st of January 2010 and the 31st of December 2016 were taken into consideration. This time frame was used because the first fully documented cases in the used registry are from 2010 and working on this evaluation started in April 2017.

From this pool of patients 351 matches (702 patients) were built to perform a matched pair analysis. Thereby, a patient with at least one tumor board in its history was matched to a patient whose case was never discussed. Further, the matched pairs had to be equal with regard to their ICD-10 (International Statistical Classification of Diseases and Related Health Problems 10) diagnosis, staging and sex. Age was matched as closely as possible. Additionally a follow-up time of at least 300 days or prior death was mandatory for all patients included.

The staging measurements used for the matching process depended on the tumor entity. TNM or UICC (Union internationale contre le cancer) classification was used for solid tumors, Durie and Salmon Score for multiple myelomas, WHO (World Health Organization) status for neurooncological tumors, Binet classification for CLL (chronic lymphocytic leukemia) and Ann Arbor for lymphomas. Along with TNM and UICC the Gleason score was used in cases with prostate carcinoma and the Clark level in cases with melanomas to further improve matching.

**Fig. 1:**
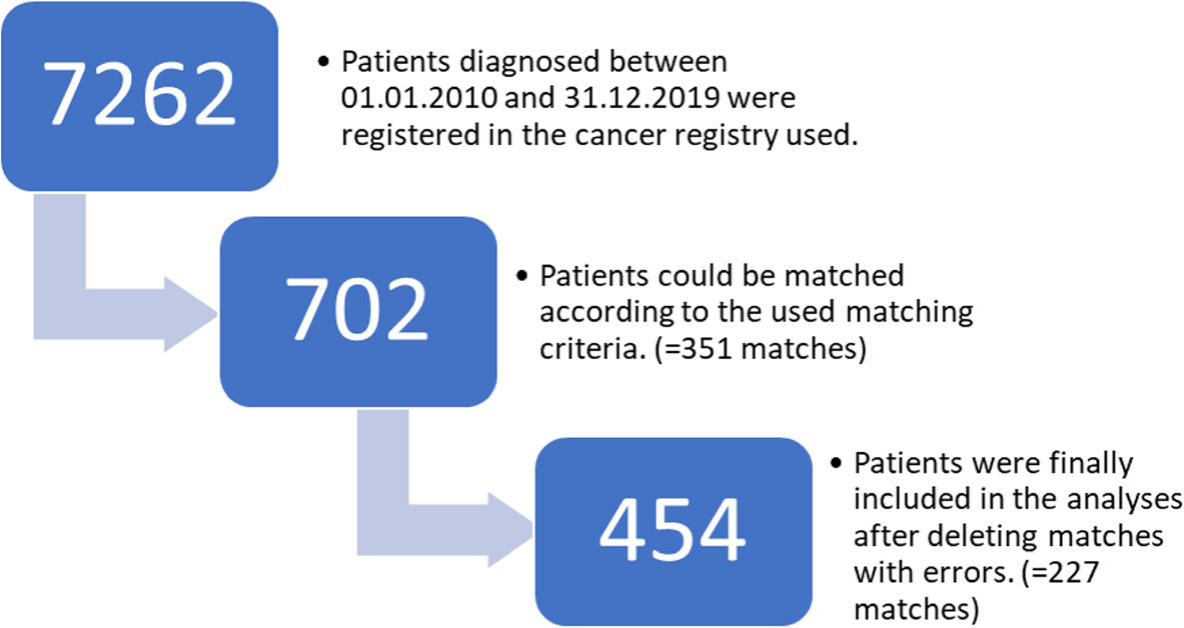
Flowchart of patient recruitment: A flowchart describing the different stages of recruitment of patients included in this analysis

All matches were checked for patients included in multiple pairs. If a patient was included in two or more matches, the match with the lowest age difference was kept while the others were deleted. In the same process the patient data was also checked for errors and missing key information as a questionable history of tumor boards, unclear staging or incomplete follow up. In case missing information could not be added, those matches were deleted. After this process, 454 patients (227 matches) were left for analysis. (Fig. 1).

### Statistical analysis

Overall survival (OS) was defined as the time between diagnosis and death. Relapse free survival (RFS) only included patients who experienced a complete remission (CR) at one point in their history and was defined as the time between diagnosis and death or relapse. Time to progression (TTP) excluded patients who died of other causes than their diagnosed cancer disease and was defined as time between diagnosis and progression or death. As measurement for the response to therapy and the tumor status the RECIST (response evaluation criteria in solid tumors) classification was used with CR (complete remission), PR (partial response), SD (stable disease) and PD (progressive disease).

The software used for all calculations and the statistical analysis was Version 23 of SPSS statistics (IBM Corp. Released 2015. IBM SPSS Statistics for Windows, Version 23.0. Armonk, NY: IBM Corp.).

The t-test for dependent samples was used for comparison of mean age and mean follow up time differences. For comparison of response to treatment and distribution of follow up time the chi square test was used. Kaplan Meyer analysis and the log rank test was used for evaluation of overall survival, relapse free survival and time to progression. *P* < 0.05 was defined as significant for all tests listed above.

### Subgroup analysis

For analysis by the number of tumor boards the cohort of patients with tumor boards was split into two sub-groups depending on the number of tumor boards in which their case was discussed. Cases of group 1 were discussed in one or two and cases of group 2 in three or more tumor boards.

For subgroup analyses depending on the tumor entity all patients included in this study were divided in a total of 15 different subgroups depending on the entity of their main tumor. The division into the subgroups leaned on the groups used in the ODS easy cancer registry and therefore they are colon and rectum carcinomas, prostate carcinomas, bronchial carcinomas, pancreatic carcinomas, malignant melanomas, non-melanotic skin cancer, gastric cancer, esophageal cancer, head and neck carcinomas, urothelial carcinomas, liver carcinomas, lymphoma, multiple myelomas, leukemia and neurooncological tumors.

## Results

### Patient characteristics

#### Age when diagnosed with cancer

The mean age of the non-tumor board cohort was 63.1 years (range 24–93) compared to 63.0 years (range 29–91) in the other cohort. The t-test for dependent samples does not show any significant differences (*p* = 0.776).

#### Gender

About 31.3% of the patients (71 matches, 142 patients total) are female while the other 68.7% are male (156 matches, 312 patients total).

#### Follow-up time

The mean follow up time in the no tumor board cohort was significantly longer with 842 days (range 5–2303) (t-test for dependent samples: *p* < 0.001) compared to 560 days (range 15–2693) in the other cohort. This result may partly be driven by the fact that in the period from 2010 to 2016 the tumor boards were certified in the University Hospital and a rising number of patients were discussed each year. Therefore 74,4% of the patients in the non tumor board cohort were diagnosed before 2015 compared to only 30.4% of the patients in the other group, which is a significant difference in distribution according to the chi square test (p < 0.001) (Table 1).

**Table 1:** The number and percentage of patients included in this study with and without multidisciplinary tumor boards in their history diagnosed each year in relation to the total number of documented tumor boards within that year. Percentages were rounded to one decimal place

Year of diagnosis	No MTB	No MTB %	No MTB %	MTB	MTB %	MTB %	Total number of MTB
2010	4	1.8%	74.4%	1	0.4%	30.4%	14
2011	8	3.5%		1	0.4%		21
2012	20	8.8%		1	0.4%		88
2013	30	13.2%		11	4.8%		126
2014	107	47.1%		55	24.2%		351
2015	54	23.8%	25.6%	124	54.6%	69.6%	478
2016	4	1.8%		34	15.0%		434
Overall	227	100%	100%	227	100%	100%	1512

#### Entities

All the 227 matched pairs were cases with a primary tumor from one of 15 different tumor entity groups. There were 4 patients with a colon or rectum carcinoma, 36 with a prostate carcinoma, 12 with a bronchial carcinoma, 48 with a pancreatic carcinoma, 36 with a malignant melanoma, 2 with non-melanotic skin cancer, 4 with gastric cancer, 2 with a esophageal carcinoma, 54 with a head and neck carcinoma, 74 with an urothelial carcinoma, 12 with a liver carcinoma, 22 with a lymphoma, 26 with multiple myelomas, 46 with leukemia and 76 with a neurooncological tumor.

### General outcome

#### Response to treatment

**Fig. 2:**
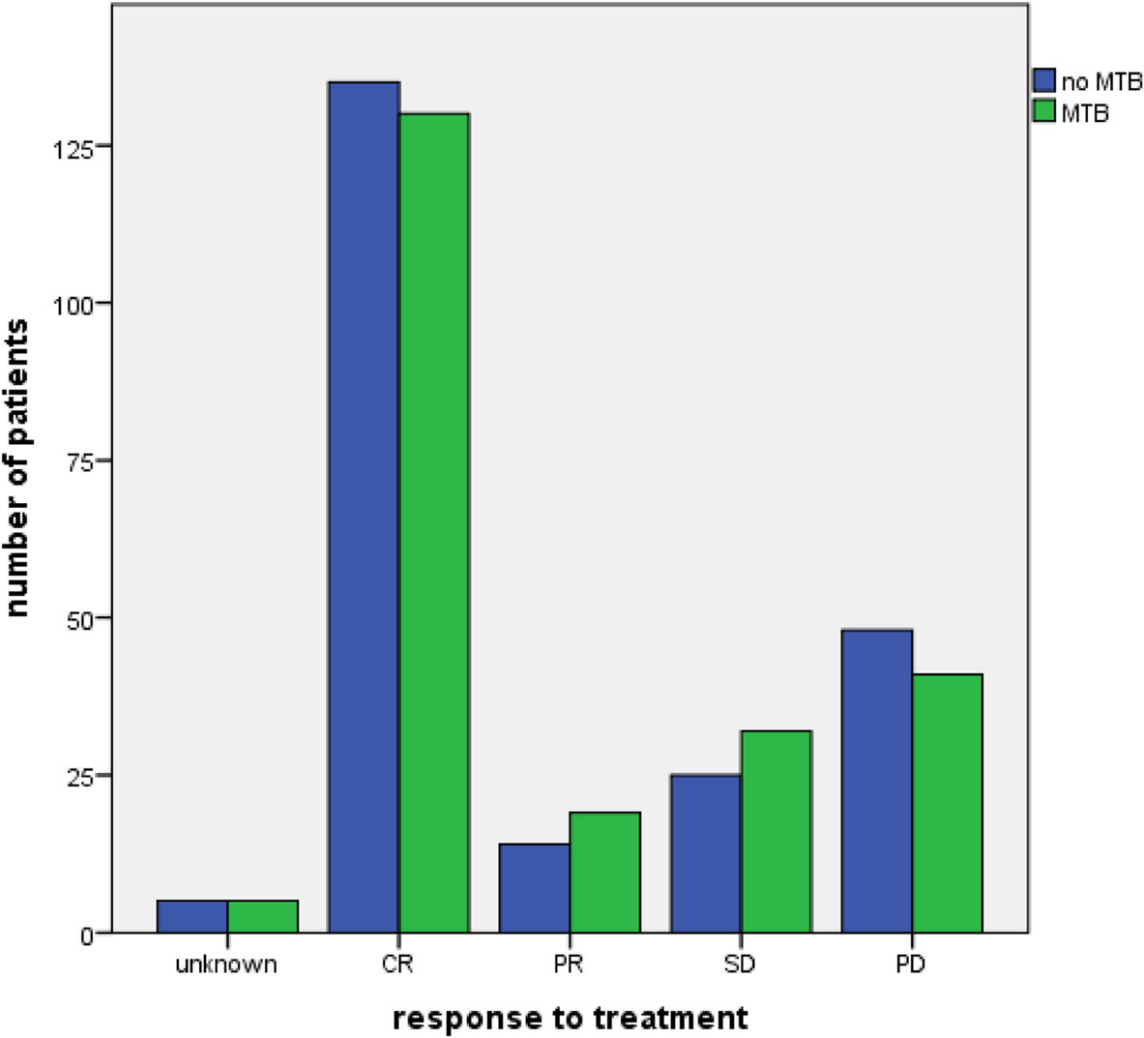
Response to treatment: Grouped bar chart of the response to treatment by RECIST criteria of patients with and without a MTB in their history (*n* = 454, chi-square = 0.688)

There was no significant difference concerning the response to treatment, as the Chi-Square test equals *p* = 0.688. While the no tumor board cohort had with 135 CR cases a few more than the other cohort with 130 CR cases, they also had with 48 compared to the 41 of the other group more PD cases. The non-tumor board cohort also had 14 PR cases and 25 SD cases, while the other group had 19 PR cases and 32 SD cases (Fig. 2).

#### Overall survival

**Fig. 3:**
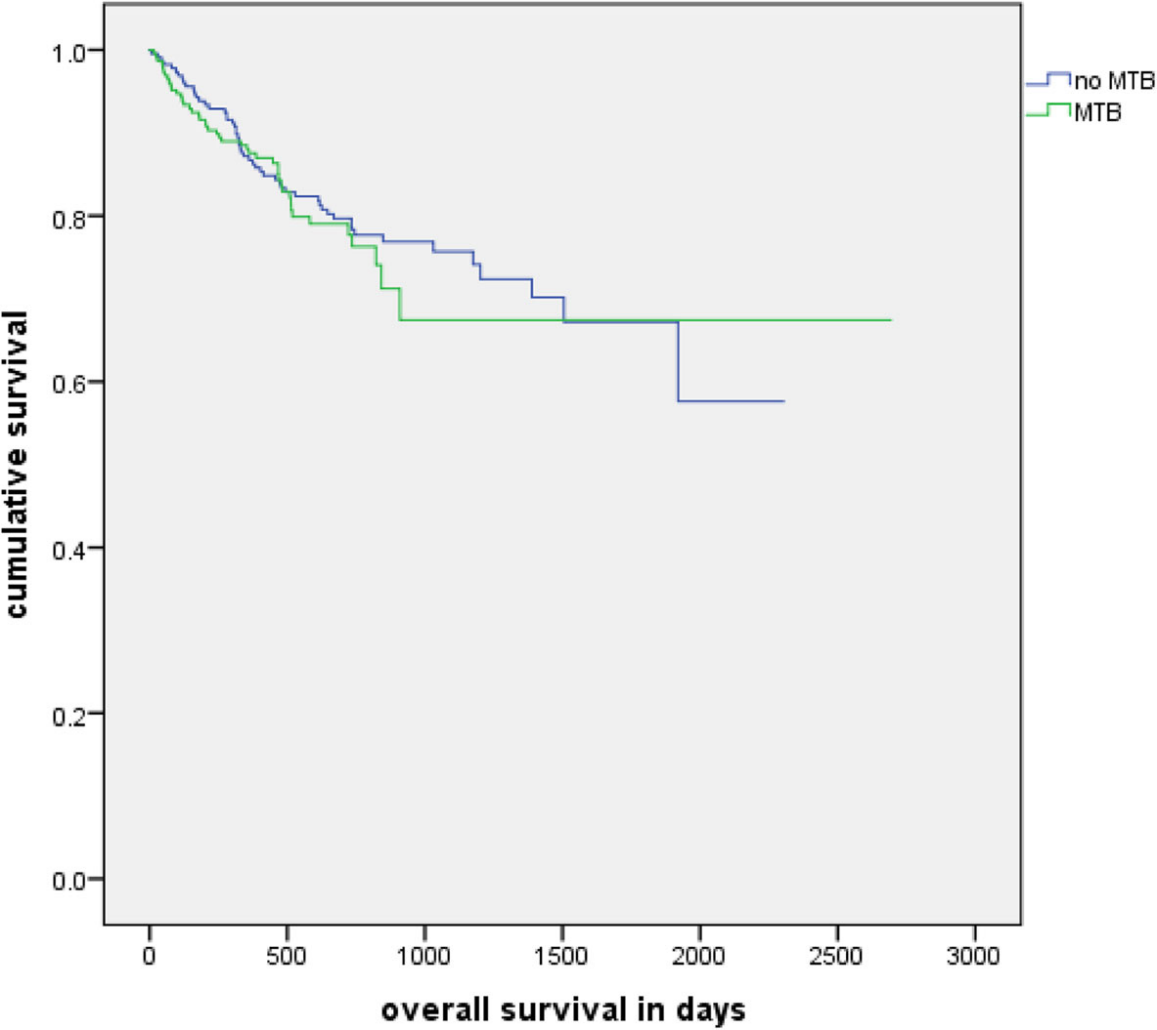
Overall survival: Kaplan-Meier analysis of the overall survival of patients with and without a MTB in their history (*n* = 454, *p* = 0.606)

The comparison of overall survival between two main cohorts did not show a significant difference (log rank, *p* = 0.606). Mean overall survival was 57 months in the no tumor board cohort and 65 months in the tumor board cohort. Furthermore, there were 54 deaths in the no tumor board cohort and 45 deaths in the other one (Fig. 3).

#### Relapse free survival

**Fig. 4:**
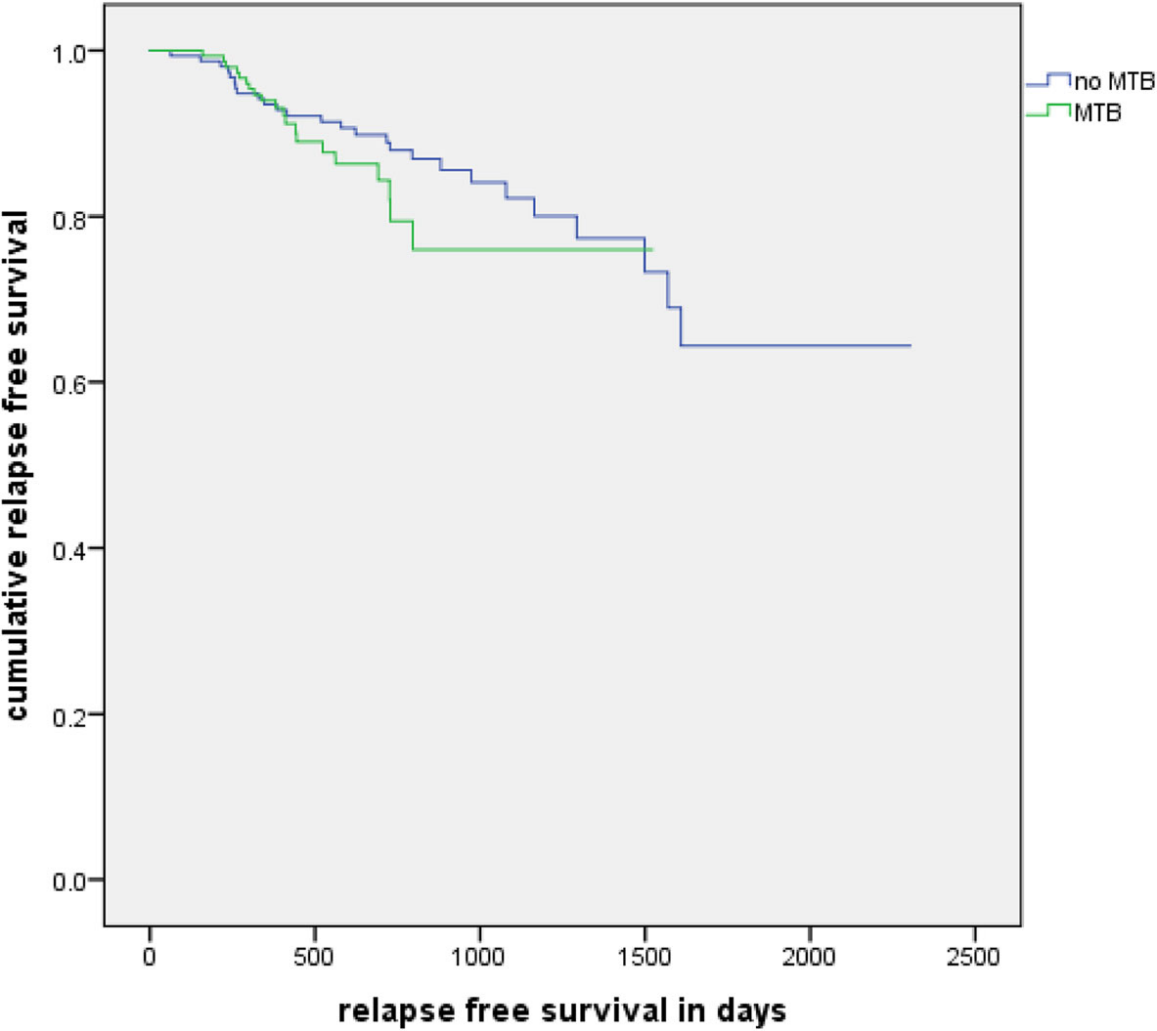
Relapse free survival: Kaplan-Meier analysis of the relapse free survival of patients with and without a MTB in their history (*n* = 304, *p* = 0.253)

There was no significant difference found. (log rank test, *p* = 0.253). Their mean RFS was 61 months, while the PFS of the tumor board cohort was 42 months. A relapse of the disease or death occurred in 26 cases of the no tumor board cohort and in 20 cases of the tumor board cohort (Fig. 4).

#### Time to progression

**Fig. 5:**
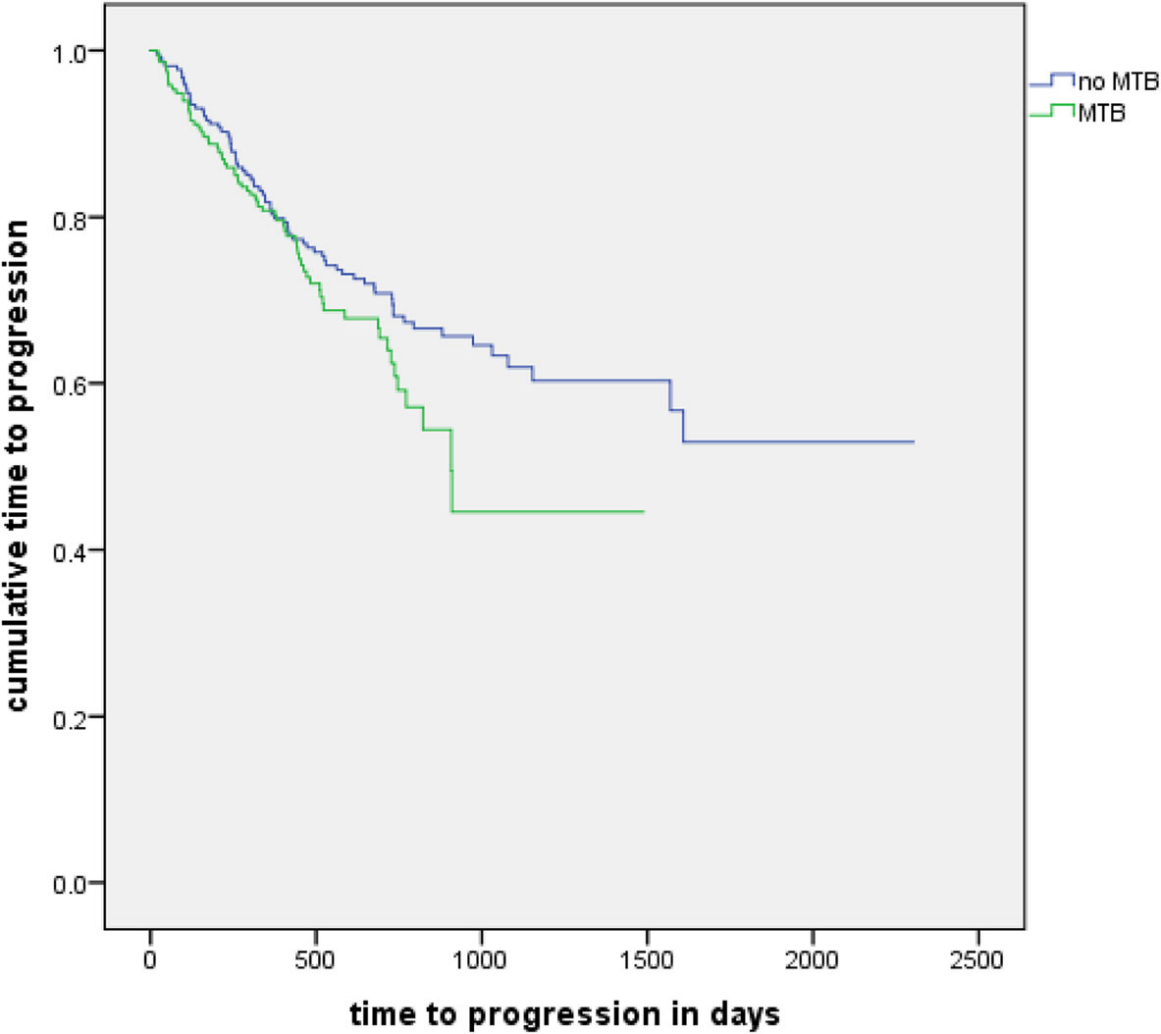
Time to progression: Kaplan-Meier analysis of the time to progression of patients with and without a MTB in their history (*n* = 429, *p* = 0.116)

No significant results (log rank test, *p* = 0.116) between the two cohorts were also found in the comparison of TTP. Mean TTP of the no tumor board cohort was 50 months and mean TTP of the tumor board cohort was 31 months. Seventy-three patients of the no tumor board cohort and 69 patients of the other cohort experienced progression or death related to their disease (Fig. 5).

### Outcome based on the number of tumor boards

With their respective matched partner group 1 with one or two MTB contained 380 and group 2 with at least 3 MTB contained 74 patients.

#### Response to treatment

Neither of the two groups showed a significant difference in the Chi square test. The results of the Chi-Square tests were *p* = 0.612 for group 1 and *p* = 0.766 for group 2.

#### Overall survival

**Fig. 6:**
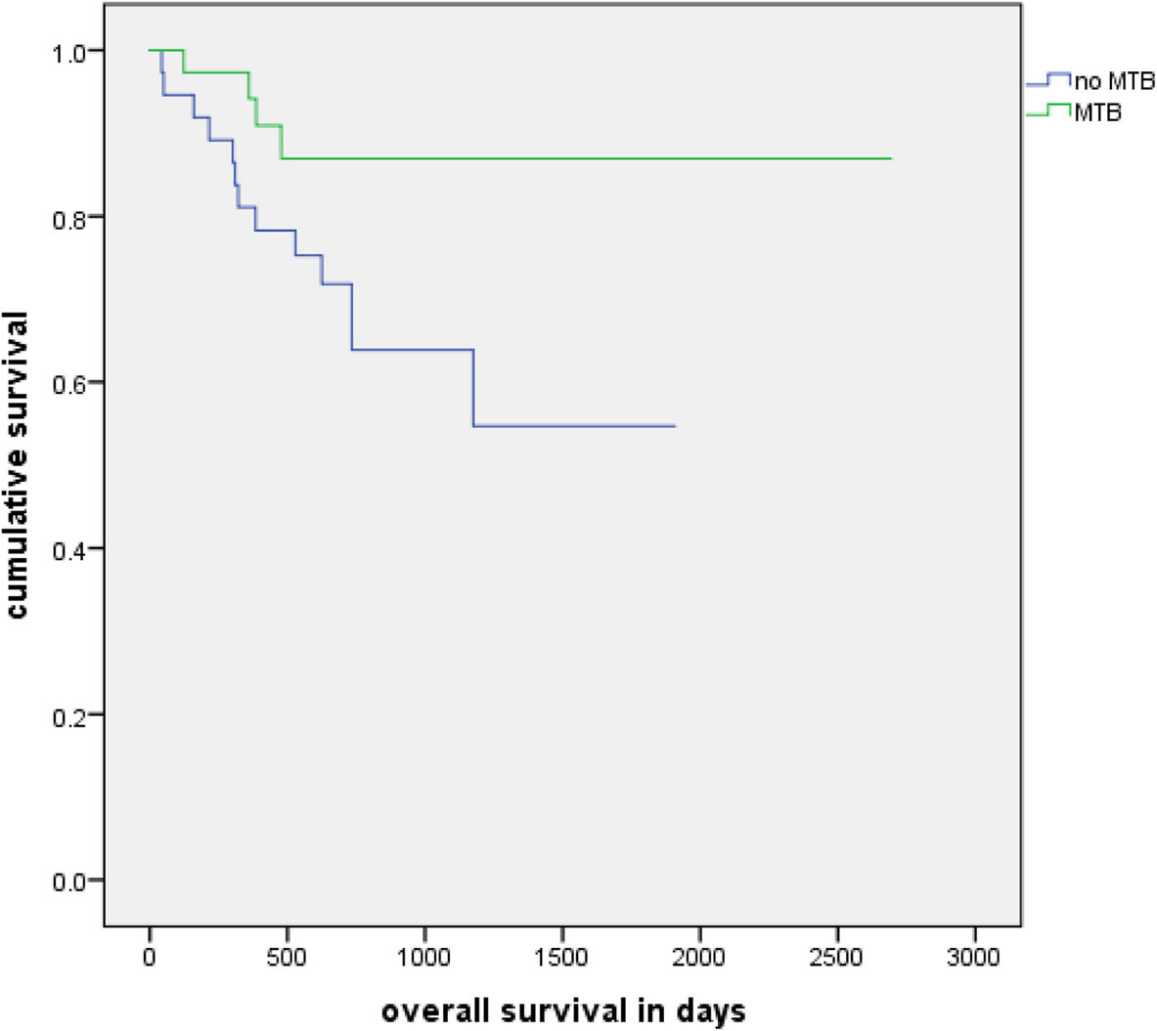
Overall survival with 3+ MTBs: Kaplan-Meier analysis of the overall survival of patients with at least 3 and their matched group without a MTB in their history (*n* = 74, *p* = 0.045)

No significant difference was found in group 1 (log rank, *p* = 0.124). In group 2, patients which were presented in at least 3 MTBs showed a significantly longer OS (log rank test, *p* = 0.045) with a mean OS of 78 months and 4 deaths in comparison to 43 months and 13 deaths (Fig. 6).

#### Relapse free survival

The log rank test revealed no significant difference in group 1 (*p* = 0.611) and group 2 (0.264).

#### Time to progression

No significant difference in the log rank test was seen in group 1 (*p* = 0.369) or group 2 (*p* = 0.171).

### Outcome based on the different tumor entities

All tumor entities with 10 or more patients were looked at separately. Thus, a subgroup analysis was made for prostate carcinoma, bronchial carcinoma, pancreatic carcinoma, malignant melanoma, head and neck carcinoma, urothelial carcinoma, liver carcinoma, lymphoma, multiple myeloma, leukemia and neurooncological tumors.

#### Response to treatment

The Chi square test did not reveal any significant differences between patients with and without an MTB in their history in any of the entities. The results in detail were *p* = 0.446 for prostate carcinomas, *p* = 0.475 for bronchial carcinomas, *p* = 0.778 for pancreatic carcinomas, *p* = 0.480 for melanomas, *p* = 0.600 for head and neck carcinomas, *p* = 0.546 for urothelial carcinomas, *p* = 0.284 for liver carcinomas, *p* = 0.449 for lymphomas, *p* = 0.700 for myelomas, *p* = 0.179 for leukemia and *p* = 0.314 for neuro oncological tumors.

#### Overall survival

None of the entities showed a significant difference between the two groups in the Kaplan Meier analysis. The results of the log rank test were *p* = 0.810 for prostate carcinomas, *p* = 0.135 for bronchial carcinomas, *p* = 0.367 for pancreatic carcinomas, *p* = 0.439 for urothelial carcinomas, *p* = 0.886 for liver carcinomas, *p* = 0.317 for lymphomas, *p* = 0.168 for myelomas, *p* = 0.470 for leukemia and *p* = 0.768 for neuro oncological tumors. There were no statistical results in melanomas and head and neck cancer, because all cases were censored.

#### Relapse free survival

There was no significant difference found in any of the included entities. The log rank test of the Kaplan Meier analysis showed *p* = 0.732 for pancreatic carcinomas, *p* = 0.202 for melanomas, *p* = 0.386 for head and neck carcinomas, *p* = 0.690 for urothelial carcinomas, *p* = 0.617 for liver carcinomas, *p* = 0.766 for lymphomas, *p* = 0.257 for myelomas, *p* = 0.945 for leukemia and *p* = 0.090 for neuro oncological tumors. All cases were censored in prostate carcinomas and bronchial carcinomas.

#### Time to progression

In the Kaplan Meier analysis none of the entities revealed a significant difference between the two groups. The results of the log rank test were *p* = 0.633 for prostate carcinomas, *p* = 0.076 for bronchial carcinomas, *p* = 0.320 for pancreatic carcinomas, *p* = 0.108 for melanomas, *p* = 0.327 for head and neck carcinomas, *p* = 0.604 for urothelial carcinomas, *p* = 0.782 for liver carcinomas, *p* = 0.167 for lymphomas, *p* = 0.285 for myelomas, *p* = 0.364 for leukemia and *p* = 0.971 for neuro oncological tumors.

## Discussion

Our analysis did not show a considerable impact of MTB on response to treatment (RTT), overall survival (OS), relapse free survival (RFS) and time to progression (TTP). However, patients with 3 or more MTBs in their history show a significant better OS than their matched group without any MTBs and patients with only one MTB have a significantly shorter OS than their matched group. Analyzing the 15 different tumor entities separately did not reveal a significant difference in RTT, OS, RFS or TTP in any of them.

A major advantage of our study is the large number of different cancer types looked at to be able to evaluate the outcome of MTB across different entity types and therefore almost in general. Also the evaluation of the outcome was broad by measuring not only the overall survival as many other studies [1, 2, 5–8, 10, 13, 14], but also the time to progression, relapse free survival and response to treatment.

On the downside all data was collected in a single center and the sample size for subgroup analysis especially by tumor entity was therefore small. The significant difference in the follow-up time is a concern too. While the number of tumor boards increased, the number of patients without a tumor board decreased consequently over the period from 2010 to 2016. As consequence, in the matching process patients diagnosed between 2015 and 2016 with a MTB were mostly matched to patients diagnosed between 2011 and 2014. The long recruitment period is also a hazard concerning possible general changes in diagnoses and treatment during this time period. Even though the adherence to MTB recommendations is comparably high with a shrinking deviance in recent years [15], it was not considered in our study and could be a factor. We matched as closely as possible by using diagnosis, staging, sex, age and a minimum follow-up time, but a pre selection bias by the treating physician to rather present severe cases in a multidisciplinary tumor board meeting cannot be totally ruled out. A general tendency to include rather simple cases with no big differences in the outcome is also possible, as complicated cases almost always get discussed in an MTB. The largest portion of the neurooncological cohort are for example meningiomas, where surgery is the treatment of choice and adjuvant therapy is usually not indicated. Also a general selection bias in patient recruitment is possible, as sufficient follow up and data was needed to include a patient which is more likely to be provided by patients with a longer survival.

Intuitively having MTB and therefore a group of experts discuss every case is the obvious choice, but the literature is not as clear yet concerning the evidence for an improved clinical outcome. While many single tumor entity group studies show an improvement in OS [1–8] some found no relevant improvement of the clinical outcome [9, 10]. Studies evaluating OS in MTB across different tumor entity groups by literature reviews [13] or comparison of centers with and without MTB [14] show weak to no evidence. Evaluation of not only survival but also relapse or progression free survival in the reviewed literature was rare and did not show evidence of an improved outcome for MTB patients [11, 12].

Our study aligns to some extent with a large portion of the literature mentioned above, as it fails at the first look to provide general evidence of an improved outcome by MTBs in RTT, RFS and TTP. To a certain extent the significantly better result of the group with 3 or more MTBs than their matched group without any shows a general positive impact of MTBs on OS though.

Furthermore, our analysis revealed an impact of the number of tumor boards. It seems to be beneficial for a patient if his case is discussed in multiple MTBs, as the OS of patients with at least three MTBs in their history is significantly better than their matched group while there is no significant difference between patients with one or two MTBs and their matched group without any MTBs. Possibly the positive effect of MTBs for a patient is exponentiated with every additional MTB discussion and review of the case. A possible hazard is the survivorship bias, as patients must be alive for longer for their case being discussed at least 3 times.

Further research seems necessary to evaluate the effect of multidisciplinary tumor boards on clinical outcome and prove their benefit. Especially a closer look on the influence of the number of tumor boards on the outcome seem to be of interest.

## Conclusion

As only one among all analysis did show a significant result, we could not find an obvious improvement of clinical outcome by MTB. However, the significantly better outcome of Patients with at least 3 MTB in their history could hint towards a positive impact of MTB on the clinical outcome of cancer patients. Specifically, a higher number of MTB per patient might increase the overall survival.

## Data Availability

The datasets used and analyzed during the current study are available from the corresponding author upon reasonable request.

## Abbreviations

ODS: Oncologic documentation system
CIO: Center for Integrated Oncology
MTB: Multidisciplinary tumor boards
RECIST: Response evaluation criteria in solid tumors
CR: Complete remission
PR: Partial remission
SD: Stable disease
PD: Progressive disease
OS: Overall survival
RFS: Relapse free survival
TTP: Time to progression
WHO: World Health Organization
UICC: Union internationale contre le cancer
CLL: Chronic lymphocytic leukemia
ICD: International Statistical Classification of Diseases and Related Health Problems
